# Inclusion of disability in primary healthcare facilities and socioeconomic inequity in Brazil

**DOI:** 10.11606/s1518-8787.2024058005634

**Published:** 2024-09-16

**Authors:** Hannah Kuper, Alexandro Rodrigues Pinto, Everton Nunes da Silva, Jorge Otávio Maia Barreto, Tim Powell-Jackson

**Affiliations:** I London School of Hygiene & Tropical Medicine. Faculty of Epidemiology and Population Health. International Centre for Evidence in Disability. London, United Kingdom; II Ministério da Saúde. Brasília, DF, Brasil; III Universidade de Brasília. Faculdade de Ceilândia. Brasília, DF, Brasil; IV Fundação Oswaldo Cruz. Brasília, DF, Brasil; V London School of Hygiene & Tropical Medicine. Faculty of Public Health and Policy. Department of Global Health and Development. London, United Kingdom

**Keywords:** Pay-for-Performance, Brazil, Disability

## Abstract

**OBJECTIVE:**

To describe disability-related performance and inequality nationwide in Brazil, and the changes that took place between 2012 and 2019 after the introduction of Programme for Improving Primary Care Access and Quality (PMAQ).

**METHODS:**

We derived scores for disability-related care and accessibility of primary healthcare facilities from PMAQ indicators collected in round 1 (2011–2013), and round 3 (2015–2019). We assessed how scores changed after the introduction of PMAQ. We used census data on *per capita* income of local areas to examine the disability-specific care and accessibility scores by income group. We undertook ordinary least squares regressions to examine the association between PMAQ scores and *per capita* income of each local area across implementation rounds.

**RESULTS:**

Disability-related care scores were low in round 1 (18.8, 95%CI 18.3–19.3, out of a possible 100) and improved slightly by round 3 (22.5, 95%CI 22.0–23.1). Accessibility of primary healthcare facilities was also poor in round 1 (30.3, 95%CI 29.8–30.8) but doubled by round 3 (60.8, 95%CI 60.3–61.3). There were large socioeconomic inequalities in round 1, with both scores approximately twice as high in the richest compared to the poorest group. Inequalities weakened somewhat for accessibility scores by round 3. These trends were confirmed through regression analyses, controlling for other area characteristics. Disability-related and accessibility scores also varied strongly between states in both rounds.

**CONCLUSIONS:**

People with disabilities are being left behind by the Brazilian healthcare system, particularly in poor areas, which will challenge the achievement of universal health coverage.

## INTRODUCTION

There are 1.3 billion people with disabilities globally^1^, including at least 17.3 people in Brazil alone^2^. People with disabilities frequently face additional difficulties in accessing healthcare, due to financial, informational, attitudinal and physical barriers. There are also concerns about the quality of care they receive^1,3^, including in Brazil. For instance, health facilities are frequently not accessible^4^, there is a lack of specialist care for people with disabilities^5^, and a lack of coordinated care for those with complex needs^6^. These discrepancies may be even larger in rural and poorer areas, and so a steep socioeconomic gradient in disability-related care is likely^7^. The right to healthcare for people with disabilities, asserted through the United Nations (UN) Convention on the Rights of Persons with Disabilities and a range of Brazilian policies and laws, may therefore be violated. Furthermore, this situation challenges the achievement of Universal Health Coverage (UHC), which, by definition, means that quality healthcare should be available for all. Healthcare services in Brazil therefore need to ensure the inclusion of people with disabilities, particularly in primary health care (PHC), which is central to the achievement of UHC.

Brazil has made strong commitments to improving PHC with the creation of a unified health system (*Sistema Único de Saúde* — SUS) in 1990^8^. SUS is a universal system that provides healthcare to the entire Brazilian population, and approximately 23% of the population are additionally covered through private insurance^9^. PHC in SUS is delivered predominantly through the Family Health Strategy, created in 1994. This strategy works through multidisciplinary Family Health Teams (FHT), including at least one physician, nurse, nurse assistant and community health agent. FHTs typically support 3 to 4 thousand people, and has a co-located Oral Health Team, consisting of one dentist and one assistant. Until 2020, groups of 4–5 Health Teams were also supported by a *Núcleo de Apoio à Saúde da Família* (NASF – Family Health Support Unit) which provided further specialist care and assistance (e.g. mental health, rehabilitation). Evaluations show that this strategy has contributed to falls in mortality, as well as reductions in racial inequalities in mortality^10–13^, deaths from cerebrovascular and heart diseases^12^, avoidable hospitalizations^14^, and socioeconomic disparities in health access^15^.

Important challenges remain for SUS, however, including persistent socioeconomic inequalities in service availability and concerns about quality of care^16^. Consequently, Brazil launched its national results-based funding program in 2011 — Programme for Improving Primary Care Access and Quality (*Programa Nacional de Melhoria do Acesso e da Qualidade da Atenção Básica* [PMAQ]). PMAQ was discontinued by the Brazilian Ministry of Health in December 2019. The aim of PMAQ was to improve access to and quality of PHC through providing financial incentives against a variety of structure, process and outcome indicators. A specific focus in the initial phase of PMAQ was to reduce socioeconomic inequalities. PMAQ was one of the largest Pay for Performance (P4P) schemes in the world, reaching around 39 thousand Family Health Teams, with an expenditure of US 1.5 billion since its inception^17^. Emerging evidence suggests that this scheme helped to reduce infant mortality^18,19^, and improved access for priority groups, such as pregnant women and children^18^, and patients with non-communicable diseases^20,21^. There has been little focus to date on whether PMAQ also serves the needs of other potentially marginalised or high-risk groups, including people with disabilities. Indeed, PMAQ, or P4P schemes more generally, could potentially worsen health disparities, as these groups may have greater healthcare needs and so providers may choose to focus on improving outcomes for the majority^22^.

There are two important aspects of inclusion of people with disabilities in the health system. First, services should be accessible for people with physical, visual, hearing and other impairments (e.g. provision of ramps, braille signage). Second, facilities should be able to provide care for disability-related conditions, such as having medication available to treat disabling mental health conditions. Both these attributes were monitored within PMAQ. The baseline national data from 2012 showed widespread gaps in accessibility of healthcare services in Brazil with rural and poorer areas faring worse^4^. However, evidence is lacking on whether accessibility improved with the introduction of PMAQs. The availability and change in disability-related care has not previously been described for Brazil.

The aim of this study is to describe disability-related performance and inequality nationwide in Brazil, and whether these measures improved between 2012 and 2019, after the introduction of PMAQ.

## METHODS

### Description of *Programa Nacional de Melhoria do Acesso e da Qualidade da Atenção Básica*

PMAQ was a voluntary national P4P programme implemented by the Brazilian SUS. The focus of PMAQ was on the performance of FHTs. Each FHT is attached to a health facility, through which it delivers individual (medical and nurse consultations, exams, vaccination, drug dispensing) and collective (epidemiological surveillance, health education) primary health care to the catchment population of 3 to 4 thousand people. In some areas (e.g. larger cities), the catchment area for a health facility can be even larger (e.g. 30 thousand plus people) and be served by several FHTs. PMAQ was rolled out over three rounds of implementation (round 1: November 2011–Mar 2013, round 2: April 2013–September 2015 and round 3: October 2015–December 2019). Each round began with an assessment of the performance of FHTs by measuring a variety of structure, process and outcome indicators. Hundreds of indicators were included (660 in round 3), which varied between rounds. Data was collected by a combination of self-assessment, routine monitoring and external evaluation. A target was set for each indicator, as well as the number of points gained if the target was met. The total scores achieved set the payment by the Ministry of Health to the municipality, which used these funds to help teams perform better, for instance by providing salaries, information, technical assistance, supplies, and improving infrastructure. Participation in PMAQ was voluntary, although the majority of municipalities joined in round 1 (71%) and this steadily increased over time (91% in round 2, 96% in round 3)^16^.

### Data Sources and Measures

Our primary measure of performance was the PMAQ indicators, which we used to derive two disability-relevant scores from indicators collected for PMAQ rounds 1 and 3:

- Disability-related care score: the full list of PMAQ indicators were reviewed to identify those related to the provision of disability-related care. Fourteen indicators were identified related to medications (e.g. for psychiatric conditions) and two specialist equipment (otoscope and ophthalmoscope). These indicators were added and scaled to derive a 0–100 disability-related care score.
- Accessibility score: external groups (experienced researchers from 45 universities) visited the PHC facilities to which the FHT were attached and undertook accessibility audits. The audit criteria varied between rounds 1 and 3, but the consistent indicators collected were whether: 1) the entrance door and corridor were wheelchair accessible, 2) interior doors and corridors were wheelchair accessible, 3) a handrail was in place, 4) a wheelchair was available. Each indicator was scored yes/no. These indicators were added and scaled to derive a 0–100 accessibility score (0 being the lowest possible score, and 100 the highest).

Data was also captured through PMAQ for each health facility on the state, municipality size, and workforce size. We used the 2010 Brazilian Population Census to derive an average monthly household income for each census area (small geographical areas with roughly 5 thousand residents). We assigned a mean household income to each FHT and health facility (PHC Unit) by linking them to the census area using geographical coordinates. We also estimated the population proportion aged 50 years and above (a strong correlate of the prevalence of disability) for each FHT and PHC Unit.

### Statistical Analysis

We described the disability-related care and accessibility scores at the level of PHC facility. The analyses focused on health facilities that took part in all 3 rounds of PMAQ to avoid selection issues. Data is presented for rounds 1 and 3, as more limited disability-related variables were collected in round 2.

We described the characteristics (e.g. region, municipality size) of the available sample of PHC facilities. The municipality size was categorized according to population (small: up 50,000 people, medium: 50,001–100,000, large: 100,001–900,000 and major cities: > 900,000). We compared the disability-related care and accessibility scores for rounds 1 and 3, calculating p-values using chi-square or *t*-test as appropriate. We then explored whether the disability-related care and accessibility scores of PHC facilities were associated with the socioeconomic characteristics of their local area, on the basis of the *per capita* income of the census sector. We divided our sample into twenty groups of equal size (i.e. 20 "ventiles") from lower to higher income, in order to describe disparities related to accessibility and disability-related care across primary health teams in terms of socioeconomic context. We then plotted the mean scores in each group (round 1, round 3, difference). We used a two-sided *t*-test to calculate 95%CI for the difference in the mean scores between the poorest and richest areas. We regressed the scores (round 1, round 3, difference), in turn, on mean *per capita* income of the census area controlling for potential confounders (census area demographics and facility characteristics), using ordinary least squares regressions. We adjusted standard errors (SEs) for clustering by census level. Finally, we examined geographical variation in scores by visualising data on the disability-related care and accessibility scores at the state level. Analyses were done in Stata 16.1.

## RESULTS

Overall, 25,085 PHC facilities were registered as participating in PMAQ. Of these, 24,625 (98%) had data available for local area income and demographics and facility characteristics, and 11,832 (48%) adhered to all three PMAQ cycles and formed our analytical sample. Sample descriptors are presented in Table 1. These PHCs were located across 11,449 census areas and served approximately 47 million people.

**Table 1:** Characteristics of analytical sample of primary healthcare facilities.

Facilities		
Number of PHC facilities		11,832
Health team by health facility		
	Mean (SD)	1.4 (0.9)
	Median (IQR)	1 (1.1)
Region		
	South-East	33%
	South	17%
	North-East	37%
	Central-West	7%
	North	6%
Municipality size		
Major (> 900,000)		7%
Large (100,001–900,000)		19%
Medium (50,001–100,000)		13%
Small (≤ 50,000)		62%
Mean (SD) workforce size		16 (10)
Graduated professionals available		75%
**Census area**		
Monthly *per capita* household income (R$)		
Mean (SD)		473 (312)
Median (IQR)		406 (261–592)
Census population older than 50 years		
Mean (SD)		19% (6%)
Median (IQR)		18% (14%–23%)

PHC: primary health care; SD: standard deviation; IQR: interquartile range.

Fourteen medications monitored in PMAQ were considered to be disability-related (eight psychiatric/mental health; four convulsions/seizure; one neurological; one combination) (Table 2). Availability of these medications was generally low in round 1 (available in 10%–21% of facilities). By round 3, availability had improved for ten medications and declined for one, but generally still remained low (12%–19%). There was also an increase in facilities reporting that they have an otoscope or ophthalmoscope between rounds 1 and 3. Overall, the disability-related care score was only 18.8 in round 1 (out of a possible 100) and improved slightly to 22·5 by round 3. Accessibility of PHC facilities was also poor in round 1, with less than half appearing to be wheelchair accessible. External accessibility, availability of a handrail and wheelchair improved between round 1 and 3, while internal accessibility worsened. The overall score (out of a possible 100) showed that mean accessibility was low in round 1 (30.3, 95%CI 29.8–30.8), but had doubled by round 3 (60.8, 95%CI 60.3–61.3).

**Table 2:** Disability-related care and accessibility indicators; round 1 and round 3.

		Round 1 n = 11,832	Round 3 n = 11,832	p-value of difference
Disability-related care indicators				
Medications				
	Phenytoin sodium	17%	16%	0.07
	Carbamazepine	18%	19%	0.007
	Chlorpromazine hydrochloride	16%	17%	0.03
	Clomipramine hydrochloride	10%	12%	< 0.001
	Amitriptyline hydrochloride	15%	17%	< 0.001
	Clonazepam	14%	17%	< 0.001
	Biperiden hydrochloride	15%	17%	< 0.001
	Haloperidol	18%	19%	0.13
	Diazepam	21%	19%	0.002
	Fluoxetine hydrochloride	14%	17%	< 0.001
	Lithium carbonate	13%	15%	< 0.001
	Phenobarbital	19%	19%	0.50
	Sodium valproate or valproic acid	13%	16%	< 0.001
	Nortriptyline hydrochloride	10%	12%	< 0.001
Otoscope available		73%^a^	88%	< 0.001
Ophthalmoscope available		14%^a^	40%	< 0.001
Mean disability-related care score (95%CI)		18.8 (18.3–19.3)	22.5 (22.0–23.1)	< 0.001
Accessibility of health facility indicators				
	Exterior wheelchair-accessible	45%	81%	< 0.001
	Interior wheelchair-accessible	31%	22%	< 0.001
	Handrail available	3%	58%	< 0.001
	Wheelchair available	42%	82%	< 0.001
	Mean accessibility score (95%CI)	30.3 (29.8–30.8)	60.8 (60.3–61.3)	< 0.001

95%CI: 95% confidence interval.

a Some missing measures.

**Figure 1:**
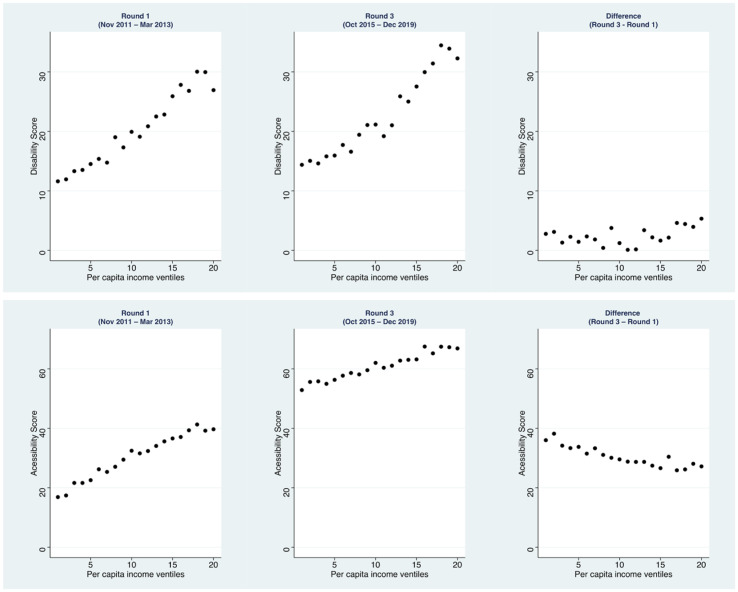
Disability-related care and accessibility scores by ventile of local area *per capita* income.

We described the disability-related care and accessibility scores in round 1 and 3 across 20 income groups, ranked from poorest (1) to richest (20; Figure 1). Disability-related care scores were positively associated with *per capita* income in round 1 and round 3. The gap was marked, for instance in round 1 it ranged from a disability-related care score of 11.6 (95%CI 10.2–13.0) in the poorest group compared to 26.9 (95%CI 24.5–29.3) in the richest group. Improvements in disability-related care scores between these rounds was modest, with no clear trend in difference in relation to income. There was also a positive correlation between accessibility score and per capita income in round 1; the gap ranged from an accessibility score of 16.9 (95%CI 15.0–18.8) in the poorest group to 39.7 (95%CI 37.2–42.2) in the richest group. By round 3, the socioeconomic gradient was still apparent although the scores had improved for all groups, and the gap between the poorest (52.9, 95%CI 50.7–55.1) and richest groups (66.9, 95%CI 64.7–69.1) had reduced. The difference in accessibility score between round 1 and 3 indicated that gains in accessibility were inversely associated with income areas.

We used regression analyses to examine the relationship between disability-related scores and accessibility scores, in turn, with census area *per capita* income, controlling for other area characteristics (Table 3). In round 1, a higher monthly *per capita* income (R$1,000) was associated with a 9.10 percentage point (95%CI 6.82–11.37) higher disability-related care score (Table 3). However, there was no evidence that this association weakened for round 3, and consequently there was no apparent change in the socioeconomic gradient over time. In round 1, accessibility scores were also worse for PHC facilities located in poorer areas. A higher monthly *per capita* income of R$1,000 (R$1,000) was associated with a 13.03 percentage point (95%CI 10.48–15.58; p < 0.0001) higher accessibility score. This association was weaker in round 3, but remained statistically significant. The change in score was negatively associated with income, indicating that the socioeconomic gradient in accessibility reduced over time.

**Table 3:** Association between disability score and accessibility, in turn, with census area income; round 1 and 3.

		Disability-related care score			Accessibility score		
		Round 1 (Nov/2011–Mar/2013)	Round 3 (Oct/2015–Dez/2019)	Difference (round 3– round 1)	Round 1 (Nov/2011–Mar/2013)	Round 3 (Oct/2015–Dez/2019)	Difference (round 3– round 1)
Characteristic							
	Monthly *per capita* income (in R$ 1,000)	9.09 (6.82 to 11.37)^c^	9.21 (6.92 to 11.50)^c^	0.12 (−1.64 to 1.88)	13.03 (10.48 to 15.58)^c^	7.06 (5.12 to 8.99)^c^	−5.97 (−8.32 to −3.63)^c^
	Proportion of census population under 5	−0.08 (−0.32 to 0.16)	−17.34 (−42.61 to 7.94)	−9.16 (−31.64 to 13.32)	−0.50 (−0.75 to −0.24)^c^	−0.22 (−0.46 to 0.02)	0.28 (−0.02 to 0.58)
	Proportion of census population over 50	0.34 (0.23 to 0.45)^c^	33.00 (21.38 to 44.62)^c^	−1.04 (−10.68 to 8.61)	−0.02 (−0.13 to 0.09)	0.18 (0.08 to 0.28)^c^	0.21 (0.08 to 0.33)^c^
Facility type (health post)							
	Others	−8.67 (−12.80 to 4.54)^c^	−5.04 (−9.37 to −0.71)^a^	3.64 (−0.12 to 7.39)	19.33 (15.04 to 23.62)^c^	50.56 (46.69 to 54.43)^c^	31.23 (26.32 to 36.14)^c^
	Health center	−5.72 (−9.80 to 1.64)^b^	−2.24 (−6.49 to 2.02)	3.48 (−0.20 to 7.16)	15.98 (11.68 to 20.28)^c^	48.54 (44.61 to 52.46)^c^	32.56 (27.62 to 37.50)^c^
	Total staff in facility	0.96 (0.89 to 1.03)^c^	1.03 (0.97 to 1.10)^c^	0.07 (0.01 to 0.13)^a^	0.67 (0.61 to 0.73)^c^	0.38 (0.33 to 0.42)^c^	−0.29 (−0.36 to −0.23)^c^
	R²	0.40	0.44	0.02	0.57	0.85	0.46
	Adjusted R²	0.40	0.44	0.02	0.57	0.85	0.46
	N° Obs.	11,832	11,832	11,832	11,832	11,832	11,832

a p < 0.05

b p < 0.01

c p < 0.001

**Figure 2:**
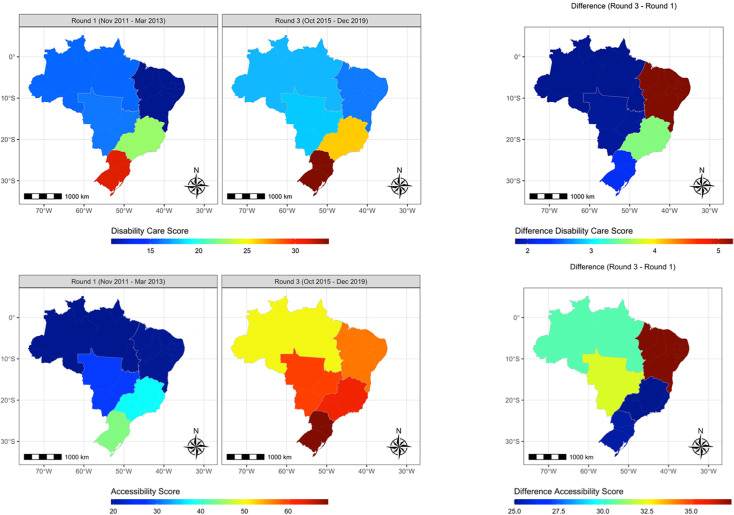
Geographic variation in disability-related and accessibility scores in round 1 and 3.

The maps highlight the variation in scores by geographic region (Figure 2). In round 1, disability-related scores appeared to be higher in the richer Southern and South-Eastern states and lowest in the poorest North and North-Eastern states. This pattern changed little between round 1 and 3. Accessibility scores also appeared generally better in the Southern states in round 1. By round 3, accessibility scores had increased across the country, but remained highest in the Southern states.

## DISCUSSION

We investigated the provision of disability-related care and accessibility in Brazil, whether there were socioeconomic inequalities in these measures, and how they changed over time after the introduction of Brazil's PMAQ in 2011. The study yielded three key findings. First, there was low availability of disability-related care and poor accessibility at the PHC level in Brazil. Second, there was a strong socioeconomic gradient in the availability of disability-related care and accessibility of facilities, with the richest areas faring twice as well as the poorest. Third, there was modest improvement of the availability of disability-related care after the introduction of PMAQ and more substantial changes in accessibility of PHC facilities. The socioeconomic gradient in accessibility attenuated somewhat after the introduction of PMAQ, but this was not observed for disability-related care.

The results indicate that PHC facilities in Brazil are not able to meet the needs of people with disabilities, and do not reach the standards set for them within PMAQ, which is also indicated by previous studies^23^. This is a violation of the right to healthcare for people with disabilities, which is enshrined in Brazilian law and policy as well as endorsed by the UN Convention on the Rights of Persons with Disabilities. Exclusion of people with disabilities from healthcare and inadequate provision of services will perpetuate the gap in health status and mortality observed for this group. Ultimately, it will become more difficult to achieve UHC and other goals if adequate provision is not made for the at least 17 million people with disabilities in Brazil, or the 1.3 billion people with disabilities globally.

The current study demonstrated a strong socioeconomic gradient in disability-related and accessibility scores. This evidence is consistent with previous analyses of overall PMAQ scores and structural quality of care scores for Brazil^15^, as well as studies focused on specific aspects of disability-related care: communication-related facilitators to accessibility (e.g. braille, hearing resources)^7^, and the presence of speech-language therapy^24^. Our analyses showed some attenuation in the socioeconomic gradient for accessibility over time, after the introduction of PMAQ. In contrast, the results from previous analyses showed a clear decline in the socioeconomic gradient of the overall PMAQ score between 2012 and 2017^15^. The findings suggest that lack of funding is a key factor perpetuating the neglect of people with disabilities in healthcare systems, and that appropriately targeted financial incentives may help to reduce the gradient. However, there is a lack of other previous studies that have assessed the socioeconomic gradient in disability-related care, particularly in low- and middle-income countries. This information would be helpful to guide policy makers and practitioners on where to invest their efforts to promote disability-inclusion and reduce inequalities.

There is a lack of evidence of what works to improve accessibility and disability-related care of PHC facilities^1^. A focus on disability within P4P programmes, such as PMAQ, may promote change in these aspects through economic incentives. The improvements in disability-related and accessibility scores after the introduction of PMAQ are consistent with P4P supporting disability-inclusion. This finding is also in line with evidence showing the effectiveness of PMAQ in Brazil, including in terms of reducing inequalities^10,12,14,15^. Additionally, within Brazil, dental speciality centers are incentivised to improve care for patients with special needs under PMAQ, and the impact of this scheme has been evaluated and showed some positive benefits^25,26^. However, it is not possible to infer causation in this observational study, in the sense that the change was because of PMAQ rather than other factors (e.g. growing awareness on disability rights). Our paper adds to the extremely sparse literature on the effectiveness of P4P, or aspects of these schemes, for people with disabilities^27^. This lack of focus on disability is observed across the literature, including a dearth of studies considering whether P4P improves value for money^28^, delivery of health interventions^29^, or disease management^30^, as well as in the design or working of P4P^31,32^. There are some exceptions, including limited data from high-income settings on the effectiveness of P4P for people with mental health conditions^27,33,34^, or dementia^35,36^. More evidence is therefore needed on the effectiveness of P4P for people with disabilities, particularly from low- and middle-income countries. More broadly, more evidence is needed on the effective interventions to improve healthcare access and quality for people with disabilities, particularly for low- and middle-income countries^1^.

There are strengths and weaknesses to the study design which must be taken into account while interpretating the results. In terms of strengths, the study used newly available data used on primary care performance for all FHT participating in PMAQ. We were able to link PHC data to fine-grained measures of socioeconomic status using census-sector level and to track the same facilities at two time points. The central limitation is that this was an observational study and so we cannot attribute impact. Moreover, PMAQ can be implemented in different ways at the municipal level, as allocation of rewards may vary (e.g. cash to teams, training). Furthermore, the indicators relevant to disability were not holistic and can be improved. The disability-related care indicators were limited in scope and had a strong focus on the provision of medication for psychiatric conditions and so ignored important aspects of care such as the presence of specific professionals who provide disability-related care (e.g. physiotherapy, speech and language). The accessibility score was also limited as it focussed only on physical accessibility of facilities, and so left out other aspects of accessibility (e.g. alternative modes of communication)^4^. Moreover, access, affordability (e.g. transport costs) or quality of care were not measured from the perspective of people with disabilities. Another concern is that disability-related care may be more available at the level of the NASF, which was not considered in these analyses. However, a requirement to attend NASF for care may create an additional layer of complexity for access for people with disabilities. Other limitations include the lack of validation of the PMAQ score as a measure of quality and it is unknown whether it is a predictor of health outcomes^15^. There are also potential concerns on the use of a crude measure of *per capita* income from the census. Generalisability of results must be considered as data was only available for a sample of PHC facilities, and included facilities delivered by teams other than FHTs (although FHT provides the vast majority of PHC in Brazil). Furthermore, PMAQ was discontinued by the Brazilian Ministry of Health in December 2019, and so the relevance and implications of our findings will need to be considered for the new funding mechanism.

## CONCLUSION

The study findings suggest that people with disabilities are being left behind by the Brazilian healthcare system, particularly in poor areas. Failure to provide accessible and holistic services will violate the right to health of people with disabilities and make it difficult for UHC to be achieved. Inclusive health services will not only improve outcomes for people with disabilities, but also for other groups (e.g. people with temporary impairments, older people). Planning for and monitoring disability inclusion will therefore create health systems that work better for all. More evidence is needed on how to promote disability inclusion, but well designed P4P programmes which include specific indicators or targets related to disability-related performance may be an option. Consulting with people with disabilities in the design of health systems will further encourage their inclusion.
